# Campbell Title Registrations to Date ‐ November 2019

**DOI:** 10.1002/cl2.1065

**Published:** 2019-12-02

1

Details of new titles for systematic reviews or evidence and gap maps that have been accepted by the Editor of a Campbell Coordinating Group are published in each issue of the journal. If you would like to receive a copy of the approved title registration form, please send an email to the Managing Editor of the relevant Coordinating Group.

## 1 EDUCATION

*School‐based language, math, and reading interventions for executive functions in children and adolescents*

Morten Kjær Thomsen, Julie Kaas Seerup, Jens Dietrichson, Martin Williams Strandby, Bjørn Christian Arleth Viinholt

30 September 2019

*Problem solving before Instruction (PS‐I) to promote learning and motivation in child and adult students*

Eduardo González‐Cabañes, Trinidad García, Catherine Chase, Jose Carlos Núñez

30 September 2019

## 2 INTERNATIONAL DEVELOPMENT

*An evidence and gap map of development evaluations conducted in India*

Ashrita Saran, Howard White, Bhumika T V, Divya Sussana Patil, Eti Rajwar

30 September 2019

*An evidence and gap map of development evaluations conducted in Karnataka 2000‐19*

Bhumika TV, Ashrita Saran, Divya Sussana Patil, Eti Rajwar, Howard White

27 September 2019

*Effectiveness of interventions to reduce environmental air pollution: An evidence and gap map in low‐ and middle‐income countries*

Judith A. Noronha, Vijay Shree Dhyani, Prathibha Kamath, Ashrita Saran, Preethy Dsouza, Anil Raj

4 November 2019

